# A population-based analysis of invasive fungal disease in haematology-oncology patients using data linkage of state-wide registries and administrative databases: 2005 - 2016

**DOI:** 10.1186/s12879-019-3901-y

**Published:** 2019-03-21

**Authors:** Jake C. Valentine, C. Orla Morrissey, Mark A. Tacey, Danny Liew, Sushrut Patil, Anton Y. Peleg, Michelle R. Ananda-Rajah

**Affiliations:** 1 Department of Infectious Diseases, Alfred Health and Central Clinical School, Monash University, Melbourne, Victoria Australia; 2 Department of Epidemiology and Preventive Medicine, Monash University, Melbourne, Victoria Australia; 3 Malignant Haematology and Stem Cell Transplantation Service, Alfred Health, Melbourne, Victoria Australia; 4 Infection and Immunity Program, Monash Biomedicine Discovery Institute, Department of Microbiology, Monash University, Clayton, Victoria Australia; 5 General Medicine Unit, Alfred Health, Melbourne, Victoria Australia; 6 Cancer Research Division, Level 13, Peter MacCallum Cancer Centre, Victorian Comprehensive Cancer Centre, 305 Grattan Street, Melbourne, Victoria 3000 Australia

**Keywords:** Invasive fungal disease, haematological malignancy, haematopoietic stem cell transplantation, data linkage, epidemiology

## Abstract

**Background:**

Little is known about the morbidity and mortality of invasive fungal disease (IFD) at a population level. The aim of this study was to determine the incidence, trends and outcomes of IFD in all haematology-oncology patients by linking Victorian hospital data to state-based registries.

**Methods:**

Episodes of IFD complicating adult haematological malignancy (HM) and haematopoietic stem cell transplantation (HSCT) patients admitted to Victorian hospitals from 1^st^ July 2005 to 30^th^ June 2016 were extracted from the Victorian Admitted Episodes Dataset and linked to the date of HM diagnosis from the Victorian Cancer Registry and mortality from the Victorian Death Index. Descriptive analyses and regression modelling were used.

**Results:**

There were 619,702 inpatient-episodes among 32,815 HM and 1,765 HSCT-patients. IFD occurring twelve-months from HM-diagnosis was detected in 669 (2.04%) HM-patients and 111 (6.29%) HSCT-recipients, respectively. Median time to IFD-diagnosis was 3, 5, 15 and 22 months in acute myeloid leukaemia, acute lymphoblastic leukaemia, Hodgkin lymphoma and multiple myeloma, respectively. Median survival from IFD-diagnosis was 7, 7 and 3 months for invasive aspergillosis, invasive candidiasis and mucormycosis, respectively. From 2005-2016, IFD incidence decreased 0.28% per 1,000 bed-days. Fungal incidence coincided with spring peaks on time-series analysis.

**Conclusions:**

Data linkage is an efficient means of evaluating the epidemiology of a rare disease, however the burden of IFD is likely underestimated, arguing for better quality hospital level surveillance data to improve management strategies.

**Electronic supplementary material:**

The online version of this article (10.1186/s12879-019-3901-y) contains supplementary material, which is available to authorized users.

## Background

Invasive fungal disease (IFD) represents a significant challenge in the management of patients with haematological malignancies (HM) undergoing cytotoxic chemotherapy and/or haematopoietic stem cell transplantation (HSCT) [1]. IFD is associated with a high mortality ranging from 29-90% [1, 2] and may affect long-term leukaemia outcomes by delaying or modifying curative chemotherapy or HSCT [3]. Few studies have evaluated the value of data linkage for IFD surveillance [4] and none have focused on the disease burden of these infections at a population-level in Victoria, Australia.

Administrative datasets are an efficient source of epidemiological data [5], yet their utility for IFD surveillance in Australia has not been well studied. The only population-based analysis of IFD in Australia used hospital discharge-coded data from 1995 to 1999 and showed that invasive candidiasis (IC) was more common than invasive aspergillosis (IA) representing 0.36% and 0.03% of all acute hospital discharges, respectively, and were associated with mortality rates between 8–26% for both IC and IA [4]. Importantly, these data predated the introduction of broad-spectrum triazole antifungal drugs that have resulted in a shift in fungal epidemiology to filamentous moulds [6] and it excluded the second most populous state in Australia, namely Victoria, with a population of 6.39 million residents [7]. The availability of state-based datasets has afforded an opportunity to revisit IFD disease burden and trends among haematology patients capturing the era of potent mould-active antifungal therapies and improvements in supportive care in cancer [8].

In this study, we linked existing population-based datasets and state registry data to characterise the epidemiology of IFD among the HM and HSCT populations across Victoria. The Victorian Admitted Episodes Dataset (VAED) is Australia’s largest hospital morbidity database and comprises demographic, administrative and clinical information coded according to the *International Statistical Classification of Diseases and Related Health Problems, Tenth Revision, Australian Modification* (ICD-10-AM) associated with every hospitalisation in Victorian public and private hospitals [9]. The Victorian Cancer Registry (VCR) has recorded all cancer diagnoses from 1982 with the exception of basal and squamous cell carcinomas of the skin in Victorian residents [10], but is only available for haematological malignancies from 1^st^ January 2008 to the 31^st^ December 2014. Overall, in-hospital and out-of-hospital mortality was evaluated with linkage to the Victorian Death Index (VDI), thus allowing comparisons of survival in patients with and without IFD. We performed data linkage between the VAED, VCR and VDI to characterise the epidemiology of IFD at a population-level over a decade in order to evaluate trends, risk-factors and to identify patient groups at high-risk for IFD.

## Methods

### Study design and setting

This was an observational, retrospective, longitudinal study of adult patients (≥16-years) diagnosed with a HM and/or post-allogeneic- (allo) or autologous (auto)-HSCT across Victorian public and private hospitals. All reporting parameters are consistent with the STrengthening the Reporting of OBservational studies in Epidemiology (STROBE) Statement [11] (Additional file 1).

### Data sources, linkage and clinical definitions

**Fig. 1:**
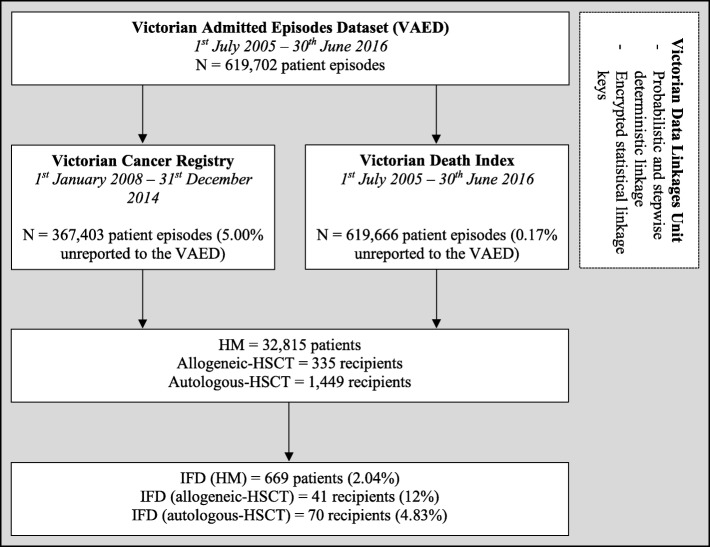
Overview of the probabilistic and stepwise deterministic linkage across administrative datasets held by the State Government of Victoria. IFD, invasive fungal disease; HM, haematological malignancy; HSCT, haematopoietic stem cell transplantation; VAED, Victorian Admitted Episodes Dataset

VAED data were linked to the dates of death from the VDI between the 1^st^ July 2005 and the 30^th^ June 2016. The datasets were linked by the Victorian Data Linkages Unit (VDLU) using probabilistic and stepwise deterministic linkage. A linkage map based on encrypted statistical linkage keys for every record across each dataset was assigned to differentiate individual patients as well as reports of multiple episode-of-care for any given patient (Fig. 1).

All patients diagnosed with a HM diagnostic- or HSCT procedural code were included. An episode-of-care was defined as a hospitalisation. After linkage of the VAED with the VCR, index hospitalisation for a HM was defined as the first appearance in an episode of an ICD-10-AM code denoting a HM (Additional file 2) appearing soon after the date of HM diagnosis as recorded in the VCR (e.g. C92, acute myeloid leukaemia (AML) appearing after the date of diagnosis from the VCR). For hospitalisations outside the dates of the VCR, i.e. before 1^st^ January 2008 or after 31^st^ December 2014, the first episode-of-care recorded in the VAED was assumed to be the index hospitalisation. A HSCT-recipient was defined by the first procedural ICD-10-AM code denoting a HSCT with the corresponding date in month and year format in the VAED.

We assessed IFD incidence as the first appearance of an ICD-10-AM code for IFD in the twelve-months from index hospitalisation for a HM-patient and twelve-months post-transplantation among the HSCT-cohort. Exclusion criteria included paediatric patients (<16-years) and HM- or HSCT-patients diagnosed with endemic or superficial fungal infections (refer to Additional file 3 for excluded codes). Cases were defined as HM or HSCT patients who had an IFD-code assigned in the first twelve-months post HM-diagnosis or HSCT and controls were defined as patients with no IFD diagnostic code. Duplicate IFD codes occurring in the same twelve-month time period were treated as the same IFD. No censoring was used when investigating the median time to IFD-onset among the HM- and HSCT-cohorts. Hospitalisation for induction chemotherapy was defined as an episode-of-care where an ICD-10-AM procedural code for chemotherapy first appeared either during or after the index hospitalisation.

### Statistical analyses

Chi-squared (*χ*^2^) and Wilcoxon rank-sum tests were used to compare the statistical significance between two categorical and non-parametric continuous covariates, respectively. A multivariable logistic regression was used to identify risk-factors for IFD since induction chemotherapy. Variables with a p<0.20 on univariate- and p<0.05 on multivariate analysis were included in a manual stepwise backward elimination process. To quantify the percent risk of IFD twelve-months from induction chemotherapy, a sigmoid function that uses marginal standardisation and prediction at the means described elsewhere was applied [12, 13]. A receiver operating curve and its C-statistic (Additional file 4) examined the model’s discrimination power and a Hosmer-Lemeshow *χ*^2^ test assessed the model’s calibration power.

Kaplan-Meier analysis and log-rank test were used to compare survival (in months) among HM-patients with and without IFD from the date of HM- and IFD-diagnosis, respectively. A time-series analysis of IFD incidence risk-adjusted for bed-day occupancy was developed. A two-sided p-value <0.05 was considered statistically significant. All statistical analyses were undertaken using Stata/SE v14.2 software (StataCorp® LLC, College Station, Texas, U.S.A.).

### Ethics

Written ethics approval was granted by the Alfred Health Human Research Ethics Committee (project number: 93/17).

## Results

### Patient characteristics

A total of 32,815 HM-patients were identified from 619,702 hospitalisations recorded in the VAED from 1^st^ July 2005 to 30^th^ June 2016. IFD occurred in 669 (2.04%) patients within twelve-months following HM-diagnosis. Among 1,765 allo- or auto-HSCT-recipients, 111 (6.29%) were diagnosed with an IFD twelve-months post-transplantation (Table 1).

**Table 1:** Demographic and Clinical Characteristics of Haematological Malignancy Patients and Haematopoietic Stem Cell Transplantation Recipients, 2005 - 2016

Characteristic	Haematological malignancy patients			Haematopoietic stem cell transplantation recipients		
	Invasive fungal disease cases, N = 669 (%)	Invasive fungal disease controls, N = 32,146 (%)	p-value	Invasive fungal disease cases, N = 111 (%)	Invasive fungal disease controls, N = 1,654 (%)	p-value
Overall crude incidence	669/32,815 = 2.04%	32,146/32,815 = 98%	< 0.001	111/1,765 = 6.29%	1,654/1,765 = 94%	< 0.001
Age (years: median [IQR])	63 [62 – 72]	71 [58 – 81]	< 0.001	54 [41 – 64]	58 [49 – 65]	0.768
15 – 25	38 (5.7)	899 (2.8)	< 0.001	12 (11)	75 (4.5)	0.011
26 – 55	187 (28)	6,407 (20)		51 (46)	714 (51)	
56 – 70	189 (29)	7,095 (22)		46 (41)	840 (51)	
71+	179 (27)	15,042 (47)		2 (1.8)	25 (14)	
Gender						
Male	406 (60)	18,364 (57)	0.065	63 (57)	1,030 (62)	0.247
Presence of neutropenia						
	549 (82)	6,735 (21)	< 0.001	107 (96)	1,478 (89)	0.018
Inpatient length of stay (LOS; days)						
Median [IQR] hospital LOS	21 [8 – 36]	1 [1 – 1]	< 0.001	32 [25 – 48]	19 [16 – 24]	< 0.001
Intensive Care Unit						
ICU admissions	37 (5.5)	585 (1.8)	< 0.001	0 (0)	4 (0.2)	1.000
Median [IQR] ICU LOS (days)	6.1 [2.8 – 16]	2.5 [1.1 – 5.3]	< 0.001	0 (0)	4.0 [1.9 – 10]	-
Mechanical ventilation (yes)	29 (4.3)	286 (0.9)	< 0.001	0 (0)	3 (0.2)	1.000
Median [IQR] ICU mechanical ventilation (days)	6.8 [2.7 – 17]	2.0 [0.6 – 6.0]	< 0.001	-	180 [82 – 280]	
Haemodialysis						
	68 (10)	478 (1.5)	< 0.001	10 (9.0)	62 (3.8)	0.007
Overall mortality						
One-month	43 (6.4)	1,983 (6.2)	0.783	0 (0)	6 (0.4)	1.000
Three-month	110 (16)	3,223 (10)	< 0.001	0 (0)	9 (0.54)	1.000
Six-month	167 (25)	4,353 (14)	< 0.001	4 (3.60)	29 (1.8)	0.149
Twelve-month	290 (43)	6,004 (19)	< 0.001	20 (18)	108 (6.5)	< 0.001
Hospital region						
Metropolitan Victoria	591 (88)	25,077 (78)	< 0.001	102 (92)	1,388 (84)	0.025
Regional Victoria	78 (12)	7,069 (22)		9 (8.11)	266 (16)	
Charlson Comorbidity Index score ≥ 4						
	192 (27)	3,808 (12)	< 0.001	27 (24)	157 (9.5)	< 0.001

*Abbreviations*: *ICU* intensive care unit, *IQR* inter-quartile range, *LOS* length of stay

### Incidence of invasive fungal disease and clinical outcomes

**Fig. 2:**
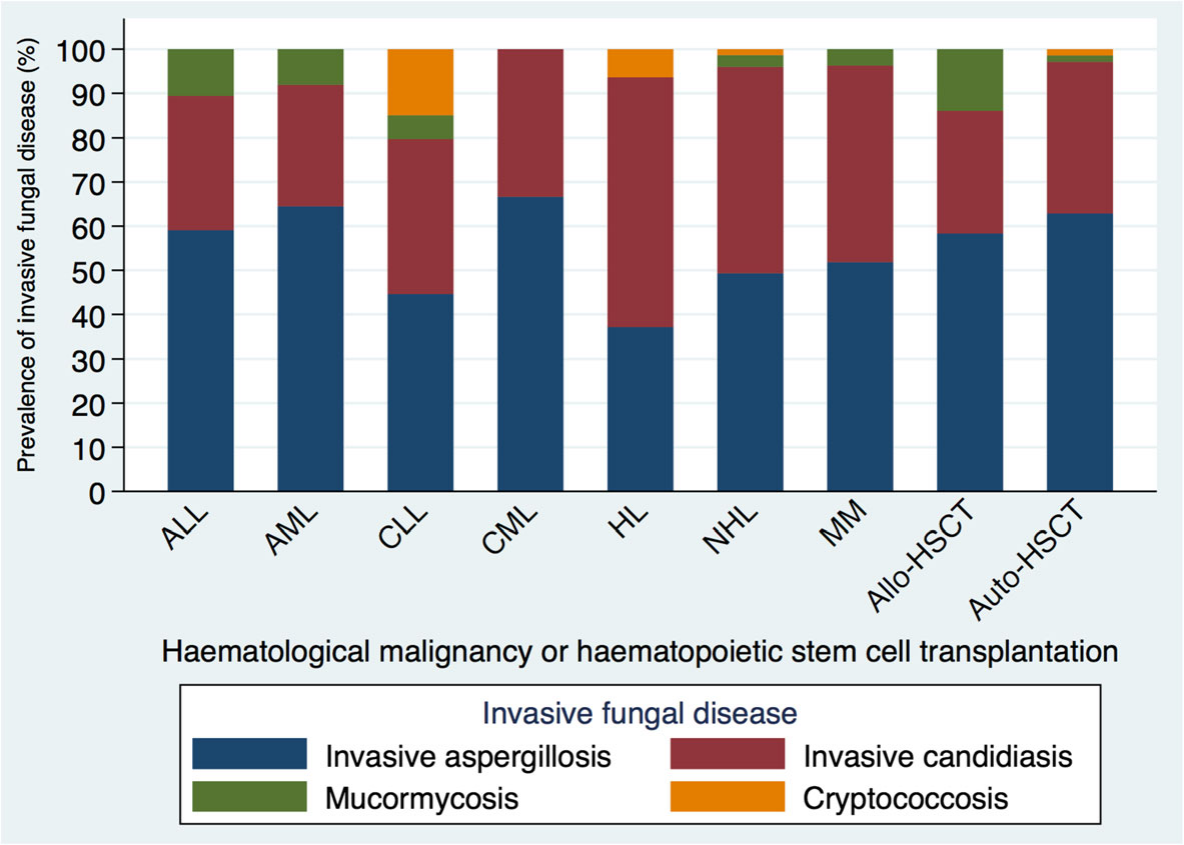
Prevalence of invasive fungal disease among cases by haematological malignancy or haematopoietic stem cell transplantation. ALL, acute lymphoblastic leukaemia; allo-HSCT, allogeneic haematopoietic stem cell transplantation; AML, acute myeloid leukaemia; auto-HSCT, autologous haematopoietic stem cell transplantation; CLL, chronic lymphocytic leukaemia; CML, chronic myeloid leukaemia; HL, Hodgkin-lymphoma; MM, multiple myeloma; NHL, non-Hodgkin lymphoma.

The distribution of IFD is presented in Table 2. The IFD incidence rate was highest in the allo-HSCT subgroup (N=48; 12%), followed by acute lymphoblastic leukaemia (ALL) (N=75; 11%), AML (N=249; 9.42%)**,** auto-HSCT-recipients (N=70; 4.83%) and aplastic anaemia (N=7,121; 1.42%). Mould diseases constituted 61% of all IFD-cases compared to 39% for yeasts (Fig. 2). IA was the most common mould disease occurring in 31 (5.07%) allo-HSCT-recipients and 29 (4.37%) AML-patients. Among the non-*Aspergillus* moulds, mucormycosis was the most prevalent, occurring in 24 (2.56%) HM-patients, with the ALL-subgroup most commonly affected (N=5; 0.75%). IC was most frequently identified in allo-HSCT-recipients (N=8; 2.38%) and ALL (N=15; 2.26%) patients.

**Table 2:** Invasive Fungal Disease Incidence Stratified by Haematological Malignancy and Haematopoietic Stem Cell Transplantation from Index Hospitalisation, 2005 – 2016

Haematological malignancy and haematopoietic stem cell transplantation	Invasive fungal disease; incidence (%)
Allogeneic-haematopoietic stem cell transplantation (N = 335)	41 (12.2)
Acute lymphoblastic leukaemia (N = 664)	75 (11)
Acute myeloid leukaemia (N = 2,644)	249 (9.42)
Autologous-haematopoietic stem cell transplantation (N = 1,449)	70 (4.83)
Aplastic anaemia (N = 7,121)	101 (1.42)
Chronic lymphocytic leukaemia (N = 3,459)	46 (1.33)
Non-Hodgkin lymphoma (N = 15,267)	192 (1.26)
Multiple myeloma (N = 5,614)	58 (1.03)
Hodgkin lymphoma (N = 2,030)	17 (0.84)
Chronic myeloid leukaemia (N = 1,240)	10 (0.81)
Other haematological malignancies (N = 1,897) ^a^	22 (1.16)

^a^Myelodysplastic syndrome patients included in ‘Other’ due to a small patient cohort.

For HM-patients, intensive care unit (ICU) admission was higher in IFD-cases (5.53%) than non-IFD controls (1.82%; p<0.001). Median length of stay in ICU was approximately 2.5-times greater in IFD-cases (6.08-days) compared to uninfected-controls (2.46-days; p<0.001). The median duration of mechanical ventilation was 3.5-times greater in IFD-cases versus non-IFD controls (6.83-days versus 1.96-days; p<0.001). A significantly greater proportion of IFD-cases required haemodialysis than controls among HM-patients (10% versus 1.49%; p<0.001) and HSCT-recipients (9.01% versus 3.75%; p=0.007), respectively. One-, three-, six- and twelve-month mortality from time of HM-diagnosis was consistently higher in IFD-cases in both HM and HSCT populations compared to controls (Table 1).

### Time to invasive fungal disease

**Fig. 3:**
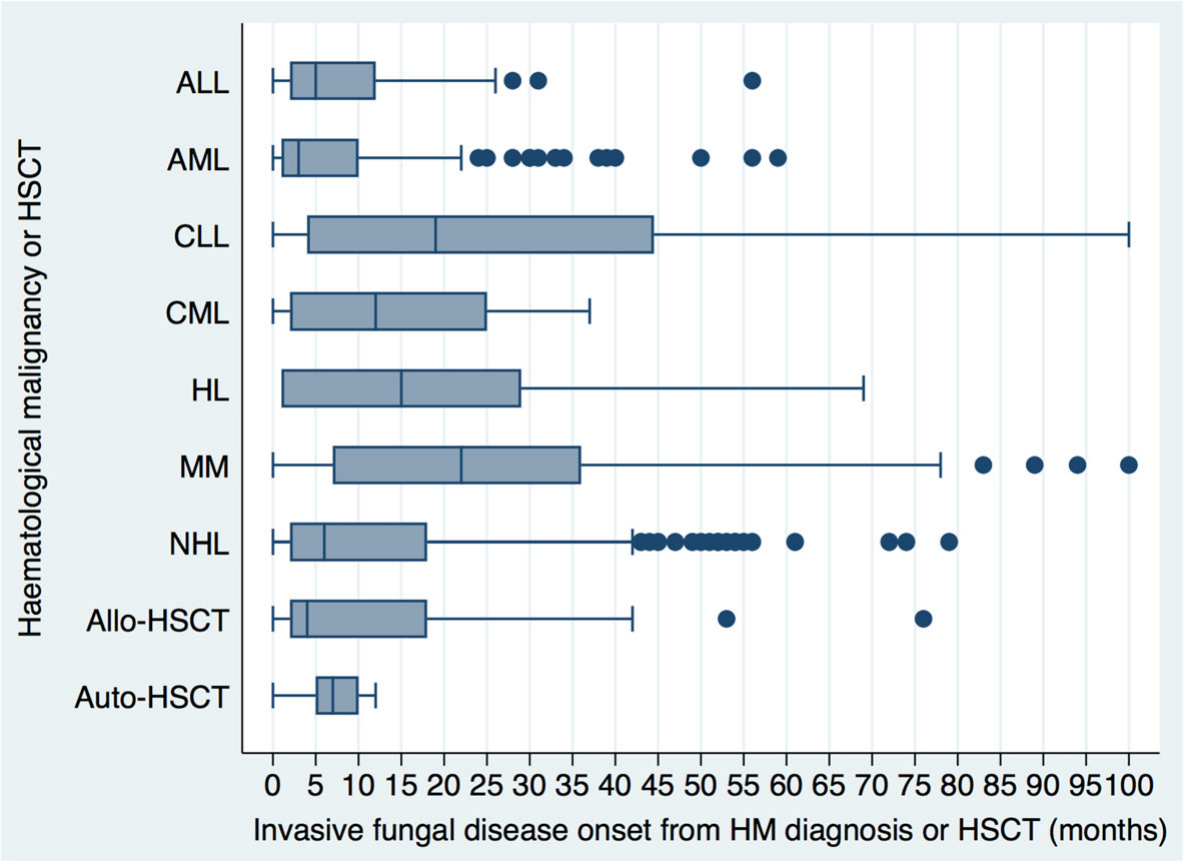
Overall distribution of time (months) to invasive fungal disease stratified by haematological malignancy and haematopoietic stem cell transplantation. ALL, acute lymphoblastic leukaemia; allo-HSCT, allogeneic-haematopoietic stem cell transplantation; AML, acute myeloid leukaemia; auto-HSCT, autologous-haematopoietic stem cell transplantation; CLL, chronic lymphoblastic leukaemia; CML, chronic myeloid leukaemia; HL, Hodgkin lymphoma; HM, haematological malignancy; IFD, invasive fungal disease; IQR, inter-quartile range; MM, multiple myeloma; NHL, non-Hodgkin lymphoma. Median [IQR] time (months) to IFD: ALL, 5 [2 – 12]; AML, 3 [1 – 10]; CLL, 19 [4 – 45]; CML, 12 [2 – 25]; HL, 15 [1 – 29]; MM, 22 [7 – 36]; NHL, 6 [2 -18]; allogeneic-HSCT, 4 [2 – 18]; autologous-HSCT, 7 [5 – 10]

The median time to IFD-onset was shortest among AML-patients (3-months) and longest for multiple myeloma (MM) patients (22-months) (Fig. 3). Of the 13 allo-HSCT-recipients with graft-versus-host-disease (GVHD), the median time to IFD was markedly shorter (1-month) compared to 91 allo-HSCT-recipients without GVHD who developed IFD (6-months) (Additional file 5).

### Survival analysis and risk of mortality

**Fig. 4:**
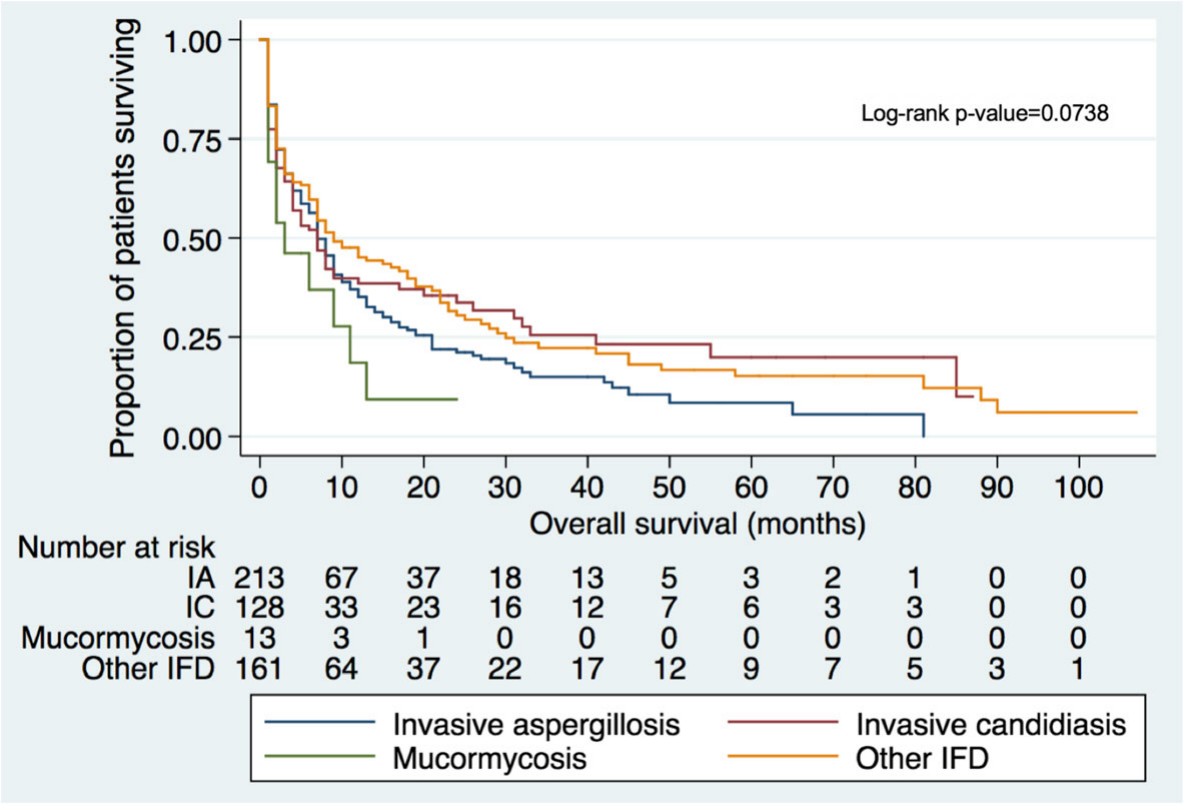
Kaplan-Meier survival curves illustrating overall survival (months) from invasive fungal disease diagnosis between invasive aspergillosis (IA), invasive candidiasis (IC), mucormycosis and other invasive fungal disease. IFD, invasive fungal disease.

The hazard ratio for IFD-onset post-HM-diagnosis was 1.24 (95% CI: 0.88–1.93; p=0.329) denoting that the instantaneous risk of mortality was 1.24-times greater when a HM-patient developed an IFD within twelve-months from index hospitalisation. After stratifying by IFD, the shortest median survival time was for mucormycosis-infected patients (median survival time: 3-months from IFD diagnosis), followed by IA and IC (7-months each) (Fig. 4).

### Time-series analysis of the risk-adjusted incidence of invasive fungal disease

**Fig. 5:**
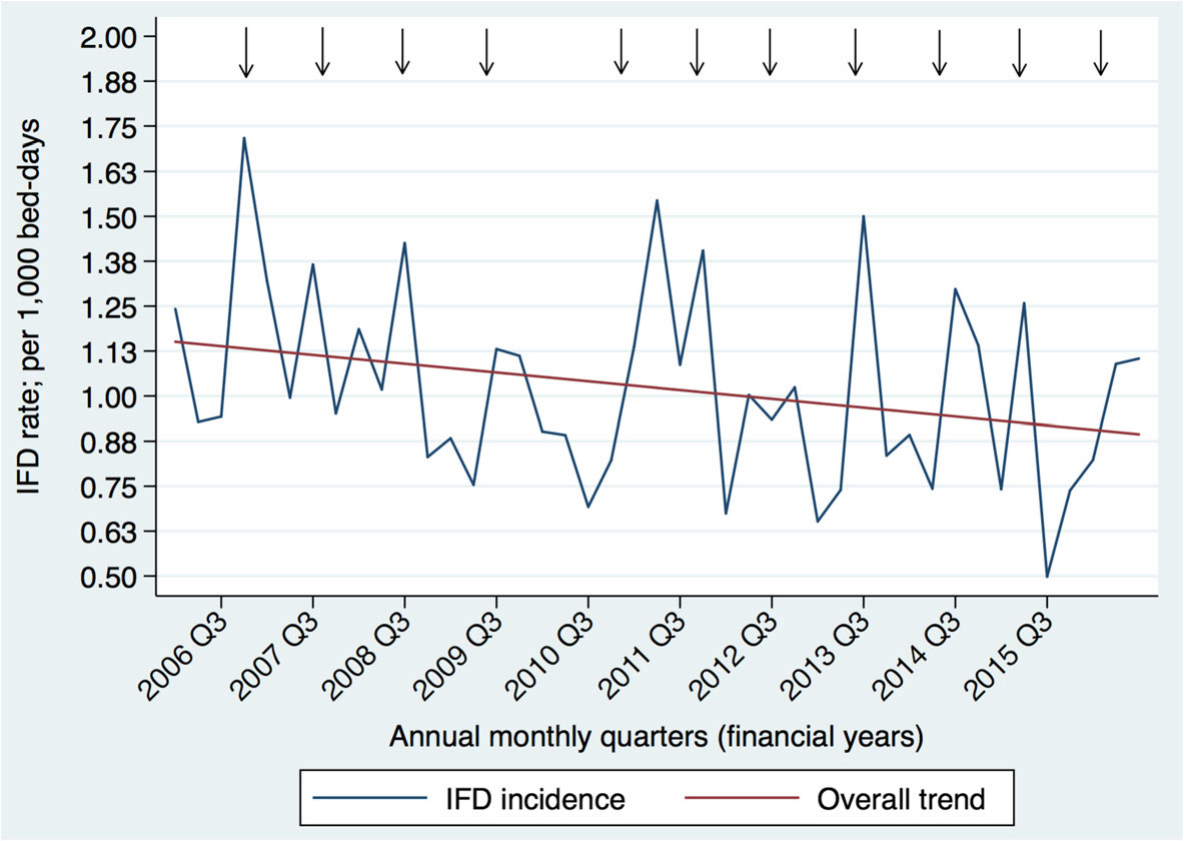
The quarterly incidence (financial years; %) of invasive fungal disease (IFD) in Victoria (2005 – 2016) along with a linear fitted values line. Black arrows indicate the spring months. Q, quarter

Over the 11-year study period, the incidence of IFD per 1,000 bed-days decreased by 0.28% (Fig. 5). A seasonal trend in IFD incidence was evident with peaks coinciding with the onset of spring (September to November in Australia).

### Risk factors for invasive fungal disease

A multivariate analysis assessing risk factors for IFD identified 10 significant covariates, including neutropenia, acute renal failure, ICU admission, residence in rural Victoria, haemodialysis, viral infection, *Clostridium difficile* infection, haematological malignancy, increasing age in years and admission to a metropolitan hospital (Table 3).

**Table 3:** Multivariate Logistic Regression Analysis of Statistically Significant Risk Factors for Invasive Fungal Diseases among Haematological Malignancy Patients Twelve-Months from Induction Chemotherapy, 2005 - 2016

Covariate	Odds ratio (OR)	Coefficient	95% confidence interval OR	p-value
Acute renal failure	1.895	0.639	1.284 – 2.798	< 0.001
Age (ten years) ^a^				
25 – 34	1.030	0.029	0.555 – 1.910	0.926
35 – 44	0.996	-0.004	0.554 – 1.790	0.988
45 – 54	1.537	0.430	0.916 – 2.580	0.103
55 – 64	1.345	0.296	0.813 – 2.225	0.249
65 – 74	1.134	0.126	0.690 – 1.864	0.619
75 – 84	0.805	-0.217	0.482 – 1.344	0.406
85 – 94	0.355	-1.037	0.186 – 0.678	0.002
95 +	0.339	-1.083	0.045 – 2.575	0.295
*Clostridium difficile* infection	2.792	1.027	1.203 – 6.480	0.017
Haematological malignancy ^a^				
ALL	11.400	2.434	5.215 – 24.918	< 0.001
AML	8.743	2.168	4.247 – 17.998	< 0.001
CLL	1.734	0.550	0.793 – 3.790	0.168
HL	0.806	-0.216	0.326 – 1.991	0.640
MDS	1.194	0.177	0.133 – 10.708	0.874
NHL	1.503	0.408	0.728 – 3.103	0.270
MM	1.221	0.199	0.570 – 2.614	0.608
Haemodialysis	3.220	1.169	1.380 – 7.510	0.007
Admission to a rural hospital ^a^	0.508	-0.676	0.368 – 0.702	< 0.001
Intensive care unit admission	1.626	0.486	1.324 – 1.997	< 0.001
Neutropenia	2.176	0.778	1.703 – 2.781	< 0.001
Region of residence ^a^				
Metropolitan Victoria	0.762	-0.271	0.596 – 0.976	0.031
Interstate	0.482	-0.730	0.273 – 0.851	0.012
Unknown	0.941	-0.061	0.278 – 3.181	0.922
Viral infection	3.117	1.137	1.081 – 8.987	0.035
CONSTANT	0.015	-4.175	-5.146 – -3.204	< 0.001

^a^Reference categories for polychotomous independent variables: Age (ten years), 15 – 24; haematological malignancy, CML; hospital region, metropolitan hospital; region of residence, rural Victoria. Reference categories were chosen as the lowest risk-burden for invasive fungal disease

*Abbreviations*: *ALL* acute lymphoblastic leukaemia, *AML* acute myeloblastic leukaemia, *CLL* chronic lymphoblastic leukaemia, *HL* Hodgkin-lymphoma, *MDS* myelodysplastic syndrome, *MM* multiple myeloma, *NHL* non-Hodgkin lymphoma, *OR* odds ratio

## Discussion

This is the first comprehensive study of IFD incidence and survival in Victoria among haematology patients over a period of 11 years and highlights the possibilities of data linkage, but also the shortcomings of administrative data for surveillance of a rare disease. The most striking finding from this study is the low overall incidence of IFD among HM-patients (2.04%) and HSCT-recipients (6.29%). It is likely that IFD is under-reported at a hospital level in coding data [14, 15] and this translates into the data generated by the VAED. Despite this shortcoming, we were able to identify periods of high-risk for a range of HMs, seasonal trends in IFD and an overall decrease in IFD incidence over the 11 years. In addition, access to a high number of clinical covariates allowed for exploration of risk-factors for IFD through multivariate regression analysis that may assist in tailoring preventative therapies like antifungal prophylaxis according to individual risk.

The epidemiological trends in IFD incidence and mortality in the HM population has historically been limited to institutional-specific reports and multicentre studies focusing predominantly on IA, IC and mucormycosis [1, 4, 16]. By contrast, through data linkage of hospital administrative data (VAED) with state-based registries (VCR and VDI), we described epidemiological trends among all HM-patients. Mould diseases predominated in keeping with global trends [6], accounting for 61% of IFD compared to 39% due to invasive candidiasis. Among mould diseases, IA was the predominant species (91%), followed by mucormycosis (8.76%); a finding concordant with recent studies [17, 18]. Mucormycosis most commonly affected allo-HSCT-recipients (1.19%), followed by ALL (0.75%) and AML (0.45%) patients and was associated with the shortest median survival time of 3-months compared to 7-months each for IA and IC. The emergence of mucormycosis as the predominant non-*Aspergillus* mould is consistent with the largest multicentre surveillance study of IFD epidemiology in HSCT recipients [6] and is likely due to several factors including longer survival post-HSCT [6, 19].

A higher IFD incidence (11%) in ALL compared to AML (9.42%) is intriguing but confirms that the ALL cohort is an emerging subgroup at high-risk of IFD with a variable fungal incidence ranging from 6.5-12% [20, 21]. Prophylaxis with azole antifungals is contra-indicated due to the drug-drug interactions with vinca alkaloids used in ALL treatment regimens [22]; but the lack of an approved standard of care from clinical trial data [21] means that clinical variation in prophylactic strategies for ALL patients is likely [23]. Patients with CLL have the third-highest IFD incidence (1.33%) and are increasingly recognised as being at high-risk of IFD due to a shift from chemo-immunotherapies to agents targeting specified B-lymphocyte pathways [24]. Indeed, IFD incidence in non-Hodgkin lymphoma (NHL) (1.26%) was the fourth highest of all HM (Table 2) which may reflect the effects of multi-agent chemotherapy in combination with immunotherapy used to treat NHL [25].

Attempts at clinical risk-stratification for IFD have been crude and restricted to broadly identifying low-, intermediate- and high-risk groups [20] in a large part because large datasets for a rare disease like IFD do not exist [26]. We confirmed risk-factors that are associated with IFD including viral infections [27], admission to a rural hospital that may reflect rural place of residence [20] and *Clostridium difficile* infection (p<0.05) which has not been previously described as a risk-factor for IFD but is prevalent in immunocompromised populations [28]. In addition, access to a high number of clinical covariates allowed exploration of a predictive tool to quantify IFD-risk at the patient-level informed by a range of risk-factors elucidated on multivariate regression analysis. We identified periods of high-risk for IFD from the time of HM diagnosis with the shortest median time seen in AML-patients (3-months) and the longest in patients with MM (22-months). The latter finding reflects the cumulative immunosuppression associated with successive lines of therapy, including immunomodulatory chemotherapies and prolonged corticosteroid exposure that is characteristic of myeloma treatment [29]. Consistent with intervals of high-risk described in Hammond *et al.* [30], risk-periods of IFD for other HMs were defined including ALL (5-months) and NHL (6-months) [20]. The shorter median time to IFD-onset after transplantation among GVHD-positive- (1-month) compared to GVHD-negative-HSCT-recipients (6-months) reflects the increased immunosuppression associated with GVHD and its treatment [31] (Additional file 5).

During the study period, there was an overall decreasing trend in IFD incidence in Victoria. The 0.28% decline in IFD incidence from 2005-2016, is contrary to the overall 3.5% increase observed in an earlier retrospective study from 1995–1999 [4]. This progressive decrease in IFD incidence is likely multifactorial and related to improved supportive care encompassing broad-spectrum antifungal prophylactic regimens for some subgroup of patients (e.g. AML, HSCT-recipients with GVHD), coupled with improved diagnostic investigations [32], clinical guidelines for IFD [33], better management of GVHD [34], cytomegalovirus prevention [27] and the introduction of high-efficiency particulate air filtration systems into some transplantation wards [35]. While an intensive diagnostic approach incorporating non-culture-based tests increases diagnostic yield [36] and corresponding fungal incidence, their availability is limited with only 35% of centres in a national Australian survey providing on-site *Aspergillus* galactomannan (GM) or polymerase chain reaction (PCR) diagnostic tests. Therefore, it seems likely that the decline in IFD incidence we observed may be explained by the uptake of mould-active prophylaxis targeting high-risk groups, as seen in a major Victorian transplant centre, which reported a reduction in IFD incidence in patients with AML from 25% with fluconazole use to 3% with posaconazole use over a 12-year period [37]. Indeed, this practice is widespread, with a nationwide survey reporting that posaconazole prophylaxis was used in 90% of AML patients undergoing chemotherapy and 68% of allogeneic-HSCT recipients, with lower rates among ALL patients of 53% [38], highlighting the lack of a standardised approach in this patient group. Consistent with the 5.7% increase in IA incidence during the warmer months as reported by Panackal *et al*. [39], the peaks in IFD incidence at the onset of spring indicates seasonality not previously described in the southern hemisphere (Fig. 5). This knowledge could ensure that preventative strategies, coupled with enhanced surveillance, also take seasonality into consideration.

Linkage of administrative and clinical datasets could potentially improve knowledge discovery for a rare disease such as IFD, but is contingent on the completeness of hospital-level data collection. Cancer surveillance systems that leverage data linkage between the VCR and clinical registries is considered a technological solution to more accurately determine the epidemiology of rare leukaemia in Victoria [40]. Limited international [14] and Australian data [15] suggest that IFD are under-reported in hospital administrative systems. This is in a large part because fungal surveillance is difficult requiring multidisciplinary input followed by adjudication of cases according to complex definitions [41]. Chang *et al.* described the poor sensitivity of coding data of 32% for proven/probable IA in HSCT-recipients and its poor positive predictive value of 15% [14]. However, the quality of coding practice is dependent on the quality of medical record documentation, particularly discharge summaries and this has been shown to be suboptimal for IFD even when fungaemia was present [15]. Institutional underreporting has implications for hospital reimbursement but also diminishes the utility of large datasets for rare disease surveillance. Furthermore, the fact that no HSCT-recipient with an IFD was admitted to the ICU in our study is implausible considering that patients with mucormycosis frequently have multiple surgeries and require ICU support (Table 1) [6]. The introduction of sensitive machine learning-based data analytics [42] could enable real-time surveillance of IFD and improve the quality of fungal reporting at the hospital level where most of these infections are managed.

There are several limitations to this study. The quality of coding data for IFD is the foremost consideration as previously discussed. Linkage of the VAED with the VCR was only available between the 1^st^ January 2008 and the 31^st^ December 2014. Thus, we relied on the VAED to identify index hospitalisations for the other years without verification against confirmed HM-diagnosis from the VCR. Secondly, as a retrospective study, our analysis is subject to misclassification or miscoding of IFD [14]. Finally, the risk-factors we identified from multivariate analysis require validation against a separate dataset, but large datasets for IFD are currently unavailable due to the lack of comprehensive surveillance systems.

## Conclusions

The true burden of IFD among haematology-oncology patients is difficult to accurately determine from hospital-based data. We hypothesise that the true incidence is likely to be higher but without implementation of surveillance systems, it will remain underestimated. The migration of hospital systems both within Victoria, and globally, to the electronic medical record provides an opportunity to improve IFD surveillance through innovative data mining techniques [42].

## Additional files

Additional file 1: STROBE Checklist. STROBE Statement and checklist of items that should be included in reports of cohort studies. (DOC 87 kb)

Additional file 2: Inclusion ICD-10-AM Codes and Invasive Fungal Disease Diagnoses Stratified by Haematological Malignancy and Haematopoietic Stem Cell Transplantation from Index Hospitalisation, 2005 – 2016. Administrative coding data used to identify invasive fungal diseases (IFD) and haematological malignancy and haemopoietic stem cell transplantation (HSCT) recipients that were included in the study. Additional file 2 also contains data on the incidence of individual IFD diagnoses stratified by underlying malignancy and HSCT. (DOCX 54 kb)

Additional file 3: : Exclusion ICD-10-AM Codes. Administrative coding data used to identify superficial fungal infections that were excluded from this study. (DOCX 14 kb)

Additional file 4: Receiver Operating Curve and C-Statistic. Receiver operating curve and its corresponding C-statistic for the evaluation of the multivariable logistic regression model. (DOCX 162 kb)

Additional file 5: Distribution of time (months) to invasive fungal disease among allogeneic haematopoietic stem cell transplantation (HSCT) recipients stratified by graft-versus-host disease (GVHD) status post-transplantation (GVHD negative, N=13; GVHD positive, N=28). Box-plot stratified by allogeneic-HSCT recipients with and without GVHD detailing the median time (in months) to invasive fungal disease onset. (DOCX 184 kb)

## Abbreviations

ALL: Acute lymphoblastic leukaemia
Allo: Allogeneic
AML: Acute myeloid leukaemia
Auto: Autologous
GM: Galactomannan
GVHD: Graft-versus-host-disease
HM: Haematological malignancy
HSCT: Haematopoietic stem cell transplantation
IA: Invasive aspergillosis
IC: Invasive candidiasis
ICD-10-AM: *International Statistical Classification of Diseases and Related Health Problems, Tenth Revision, Australian Modification*
ICU: Intensive care unit
IFD: Invasive fungal disease
MM: Multiple myeloma
NHL: Non-Hodgkin lymphoma
PCR: Polymerase chain reaction
STROBE: STrengthening the Reporting of OBservational studies in Epidemiology
VAED: Victorian Admitted Episodes Dataset
VCR: Victorian Cancer Registry
VDI: Victorian Death Index
VDLU: Victorian Data Linkages Unit
