# Supplementary material for: A population-based analysis of invasive fungal disease in haematology-oncology patients using data linkage of state-wide registries and administrative databases: 2005 - 2016
Source: BMC Infect Dis. 2019 Mar 21;19:274. doi: 10.1186/s12879-019-3901-y (PMC6429824; doi:10.1186/s12879-019-3901-y)
Supplement: Supplementary file 2 — Inclusion ICD-10-AM Codes and Invasive Fungal Disease Diagnoses Stratified by Haematological Malignancy and Haematopoietic Stem Cell Transplantation from Index Hospitalisation, 2005 – 2016. Administrative coding data used to identify invasive fungal diseases (IFD) and haematological malignancy and haemopoietic stem cell transplantation (HSCT) recipients that were included in the study. Additional file 2 also contains data on the incidence of individual IFD diagnoses stratified by underlying malignancy and HSCT. (DOCX 54 kb) [file 12879_2019_3901_MOESM2_ESM.docx]

**Additional File 2**

**Table 4. Invasive Fungal Disease Diagnoses Stratified by Haematological Malignancy and Haematopoietic Stem Cell Transplantation from Index Hospitalisation, 2005 - 2016**

|  | **Number of total haematological malignancy patients (N); incidence (%)** | | | | | | | | **HSCT (N); incidence (%)** | |
| --- | --- | --- | --- | --- | --- | --- | --- | --- | --- | --- |
| ***Invasive fungal disease* ^a^** | **ALL, N = 664** | **AML, N = 2,644** | **CLL, N = 3,459** | **CML, N = 1,240** | **HL, N = 2,030** | **NHL, N = 15,267** | **MM, N = 5,614** | **Other, N = 1,897 ^b^** | **Allogeneic HSCT; N = 335** | **Autologous HSCT; N = 1,449** |
| ***Invasive fungal disease diagnoses (n)*** | | | | | | | | | | |
| **Invasive aspergillosis (n = 302; 32%)** | 29 (4.37) | 99 (3.74) | 15 (0.43) | 4 (0.32) | 6 (0.30) | 71 (0.47) | 16 (0.29) | 14 (0.74) | 17 (5.07) | 31 (2.39) |
| **Candidiasis of other sites (n = 108; 12%)** | 9 (1.36) | 23 (0.87) | 8 (0.23) | 1 (0.08) | 6 (0.30) | 32 (0.21) | 7 (0.12) | 8 (0.42) | 6 (1.79) | 8 (0.55) |
| **Candidal sepsis (n = 92; 9.80%)** | 5 (0.75) | 19 (0.72) | 4 (0.12) | 1 (0.08) | 3 (0.15) | 35 (0.23) | 7 (0.12) | 7 (0.37) | 2 (0.60) | 9 (0.62) |
| **Mucormycosis (n = 29; 3.09%)** | 5 (0.75) | 12 (0.45) | 2 (0.06) | 0 (0) | 0 (0) | 4 (0.03) | 1 (0.02) | 0 (0) | 4 (1.19) | 1 (0.07) |
| **Cryptococcosis (n = 9; 1.00%)** | 0 (0) | 0 (0) | 5 (0.14) | 0 (0) | 1 (0.05) | 2 (0.01) | 0 (0) | 0 (0) | 0 (0) | 1 (0.07) |
| **Candidal endocarditis (n= 2; 0.21%)** | 1 (0.15) | 0 (0) | 0 (0) | 0 (0) | 0 (0) | 0 (0) | 0 (0) | 1 (0.05) | 0 (0) | 0 (0) |
| **Chromomycosis and phaeomycotic abscess (n = 1; 0.11%)** | 0 (0) | 0 (0) | 1 (0.03) | 0 (0) | 0 (0) | 0 (0) | 0 (0) | 0 (0) | 0 (0) | 0 (0) |
| **Pneumocystosis (n = 1; 0.11%)** | 0 (0) | 0 (0) | 0 (0) | 0 (0) | 1 (0.05) | 0 (0) | 0 (0) | 0 (0) | 0 (0) | 0 (0) |
| **Candidal meningitis (n = 0; 0%)** | 0 (0) | 0 (0) | 0 (0) | 0 (0) | 0 (0) | 0 (0) | 0 (0) | 0 (0) | 0 (0) | 0 (0) |
| **Mycetoma (n = 0; 0%)** | 0 (0) | 0 (0) | 0 (0) | 0 (0) | 0 (0) | 0 (0) | 0 (0) | 0 (0) | 0 (0) | 0 (0) |
| **Sporotrichosis (n = 0; 0%)** | 0 (0) | 0 (0) | 0 (0) | 0 (0) | 0 (0) | 0 (0) | 0 (0) | 0 (0) | 0 (0) | 0 (0) |
| **Other IFD NEC (n = 212; 23%)** | 26 (3.92) | 93 (3.52) | 9 (0.26) | 3 (0.24) | 5 (0.25) | 30 (0.20) | 11 (0.20) | 6 (0.32) | 12 (3.58) | 17 (1.17) |
| **Unspecified IFD / fungaemia (n = 183; 19%)** | 29 (4.37) | 61 (2.31) | 16 (0.46) | 5 (0.40) | 6 (0.30) | 21 (0.14) | 15 (0.27) | 15 (0.79) | 7 (2.00) | 8 (0.55) |
| **TOTAL invasive fungal disease diagnoses (n=939)** | 104 (16) | 307 (12) | 60 (1.73) | 14 (1.13) | 28 (1.38) | 195 (1.28) | 60 (1.07) | 51 (2.69) | 48 (14) | 75 (5.18) |

^a^ Invasive fungal diseases listed in the order of highest to lowest incidence across the haematological malignancy populations. Other and unspecified invasive fungal disease listed last

^b^ Myelodysplastic syndrome patients supplemented with ‘Other’ due to a small patient cohort.

Abbreviations: ALL, acute lymphoblastic leukaemia; AML, acute myeloid leukaemia; CLL, chronic lymphoblastic leukaemia; CML, chronic myeloid leukaemia; HL, Hodgkin lymphoma; HSCT, haematopoietic stem cell transplantation; IFD, invasive fungal disease; NEC, not elsewhere classified; NHL, non-Hodgkin lymphoma; MM, multiple myeloma

**Table 5. Univariate Logistic Regression Analysis of Statistically Significant Risk Factors for Invasive Fungal Diseases among Haematological Malignancy Patients Twelve-Months from Induction Chemotherapy, 2005 - 2016**

| **Covariate** | **Odds ratio (OR)** | **Coefficient** | **95% confidence interval OR** | **p-value** |
| --- | --- | --- | --- | --- |
| **Acute renal failure** | 5.286 | 1.665 | 3.922 – 7.122 | < 0.001 |
| **Age (ten years) ^a^** |  |  |  |  |
| **25 – 34** | 0.702 | -0.354 | 0.412 – 1.196 | 0.193 |
| **35 – 44** | 0.663 | -0.411 | 0.406 – 1.081 | 0.099 |
| **45 – 54** | 0.894 | -0.113 | 0.586 – 1.362 | 0.601 |
| **55 – 64** | 0.745 | -0.294 | 0.497 – 1.112 | 0.154 |
| **65 – 74** | 0.616 | -0.484 | 0.413 – 0.918 | 0.017 |
| **75 – 84** | 0.348 | -1.056 | 0.230 – 0.526 | < 0.001 |
| **85 – 94** | 0.139 | -1.975 | 0.081 – 0.238 | < 0.001 |
| **95 +** | 0.099 | -2.317 | 0.013 – 0.727 | 0.023 |
| **Bacterial infection** | 8.122 | 2.095 | 6.685 - 9.869 | < 0.001 |
| **Bacterial pneumonia** | 7.661 | 2.036 | 3.619 – 16.218 | < 0.001 |
| **Bronchiolitis obliterans organising pneumonia** | 23.749 | 3.168 | 2.151 – 262.185 | 0.010 |
| ***Clostridium difficile* infection** | 7.661 | 2.036 | 3.619 – 16.218 | < 0.001 |
| **Cytomegaloviral serostatus positive** | 7.314 | 2.000 | 1.648 – 32.470 | < 0.001 |
| **Delirium** | 5.867 | 1.769 | 3.352 – 10.268 | < 0.001 |
| **Gender ^a^** | 0.830 | -0.186 | 0.714 - 0.965 | 0.015 |
| **Haematological malignancy ^a^** |  |  |  |  |
| **ALL** | 17.410 | 2.857 | 8.775 – 34.541 | < 0.001 |
| **AML** | 11.639 | 2.454 | 6.166 – 21.973 | < 0.001 |
| **CLL** | 1.573 | 0.453 | 0.784 – 3.155 | 0.202 |
| **HL** | 1.065 | 0.063 | 0.477 – 2.377 | 0.879 |
| **MDS** | 1.207 | 0.188 | 0.153 – 9.154 | 0.859 |
| **NHL** | 1.666 | 0.511 | 0.881 – 3.150 | 0.116 |
| **MM** | 1.330 | 0.285 | 0.680 – 2.600 | 0.405 |
| **Haemodialysis** | 7.698 | 2.041 | 4.167 – 14.222 | < 0.001 |
| **Heart failure** | 2.891 | 1.062 | 1.607 – 5.201 | < 0.001 |
| **Hepatic failure** | 6.823 | 1.920 | 2.892 – 16.101 | < 0.001 |
| **Admission to a rural hospital ^a^** | 0.488 | -0.717 | 0.391 – 0.610 | < 0.001 |
| **Intensive care unit admission** | 1.958 | 0.672 | 1.659 – 2.311 | < 0.001 |
| **Length of stay** $\boldsymbol{\geq}$ **21 days** | 2.443 | 0.893 | 2.076 – 2.875 | < 0.001 |
| **Neutropenia** | 9.450 | 2.246 | 7.972 – 11.202 | < 0.001 |
| **Neutropenic enterocolitis** | 6.322 | 1.844 | 4.740 – 8.432 | < 0.001 |
| **Region of residence ^a^** |  |  |  |  |
| **Metropolitan Victoria** | 1.185 | 0.170 | 1.001 – 1.403 | 0.048 |
| **Interstate** | 0.865 | -0.145 | 0.537 – 1.392 | 0.550 |
| **Unknown** | 2.176 | 0.777 | 0.792 – 5.975 | 0.131 |
| **Respiratory failure** | 5.566 | 1.717 | 3.245 – 9.547 | < 0.001 |
| **Total parenteral nutrition** | 7.035 | 1.951 | 3.913 – 12.648 | < 0.001 |
| **Upper respiratory tract infection** | 9.059 | 2.204 | 4.591 – 17.877 | < 0.001 |
| **Urinary tract infection** | 2.997 | 1.098 | 1.665 – 5.395 | < 0.001 |
| **Viral infection** | 17.427 | 2.858 | 7.733 – 39.273 | < 0.001 |
| **Viral pneumonia** | 8.288 | 2.115 | 2.859 – 24.027 | < 0.001 |

^a^ Reference categories for polychotomous independent variables: Age (ten years), 15 – 24; gender, male; haematological malignancy, CML; hospital region, metropolitan hospital; region of residence, rural Victoria. Reference categories were chosen as the lowest risk-burden for invasive fungal disease

Abbreviations: ALL, acute lymphoblastic leukaemia; AML, acute myeloblastic leukaemia; CLL, chronic lymphoblastic leukaemia; HL, Hodgkin-lymphoma; MDS, myelodysplastic syndrome; MM, multiple myeloma; NHL, non-Hodgkin lymphoma; OR, odds ratio

Lower respiratory tract infection and bronchoscopy were omitted from the logistic regression model to avoid introducing confounding bias as these are known factors associated with IFD progression.

**Table 6. Haematological Malignancy Category Definitions and its Corresponding ICD-10-AM Diagnostic Code, Eighth Edition.**

| **Haematological malignancy** | **Definition(s)** | **ICD-10-AM diagnostic code** | **Modifications to ICD-10-AM diagnostic codes from fourth to seventh editions** |
| --- | --- | --- | --- |
| **Acute lymphoblastic leukaemia** | Acute lymphoblastic leukaemia (without/in remission) | C910 (0/1) | - |
| **Acute myeloblastic leukaemia** | Acute myeloblastic leukaemia (without/in remission) | C920 (0/1) | - |
|  | Acute myelomonocytic leukaemia (without/in remission) | C925 (0/1) | - |
|  | Acute myeloid leukaemia with 11q23-abnormality (without/in remission) | C926 (0/1) | - |
|  | Acute myeloid leukaemia with multilineage dysplasia (without/in remission) | C928 (0/1) | - |
| **Chronic lymphocytic leukaemia** | Chronic lymphoblastic leukaemia of B-cell type (without/in remission) | C911 (0/1) | C912 (0/1) |
| **Chronic myeloid leukaemia** | Chronic myeloid leukaemia, BCR/ABL-positive | C921 (0/1) | - |
| **Hodgkin lymphoma** | Nodular lymphocyte predominant Hodgkin lymphoma | C810 | - |
|  | Nodular sclerosis (classical) Hodgkin lymphoma | C811 | - |
|  | Mixed cellularity (classical) Hodgkin lymphoma | C812 | - |
|  | Lymphocyte depleted (classical) Hodgkin lymphoma | C813 | - |
|  | Lymphocyte-rich (classical) Hodgkin lymphoma | C814 | - |
|  | Other (classical) Hodgkin lymphoma | C817 | - |
|  | Hodgkin lymphoma, unspecified | C819 | - |
| **Non-Hodgkin lymphoma** | Follicular lymphoma grade 1 | C820 | - |
|  | Follicular lymphoma grade 2 | C821 | - |
|  | Follicular lymphoma grade 3, unspecified | C822 | - |
|  | Follicular lymphoma grade 3a | C823 | - |
|  | Follicular lymphoma grade 3b | C824 | - |
|  | Diffuse follicle centre lymphoma | C825 | - |
|  | Cutaneous follicle centre lymphoma | C826 | - |
|  | Other types of follicular lymphoma | C827 | - |
|  | Follicular lymphoma, unspecified | C829 | - |
|  | Small cell B-cell lymphoma | C830 | - |
|  | Mantle cell lymphoma | C831 | - |
|  | Diffuse large B-cell lymphoma | C833 | - |
|  | Lymphoblastic (diffuse) lymphoma | C835 | - |
|  | Burkitt lymphoma | C837 | - |
|  | Other non-follicular lymphoma | C838 | - |
|  | Non-follicular (diffuse) lymphoma, unspecified | C839 | - |
|  | Mycosis fungoides | C840 | - |
|  | Sézary disease | C841 | - |
|  | Peripheral T-cell lymphoma, NEC | C844 | - |
|  | Anaplastic large cell lymphoma, ALK-positive | C846 | - |
|  | Anaplastic large cell lymphoma, ALK-negative | C847 | - |
|  | B-cell lymphoma, unspecified | C851 | - |
|  | Mediastinal (thymic) large B-cell lymphoma | C852 | - |
|  | Other specified types of non-Hodgkin lymphoma | C957 | - |
|  | Non-Hodgkin lymphoma, unspecified | C859 | - |
|  | Waldenström macroglobulinaemia (without/in remission) | C880 (0/1) | - |
|  | MALT-lymphoma (without/in remission) | C884 (0/1) | - |
| **Multiple myeloma** | Multiple myeloma (without/in remission) | C900 (0/1) | - |
| **Myelodysplastic syndrome** | Myelodysplastic syndrome | D46 | D47.4 |
|  | Myelodysplastic syndrome and myeloproliferative disease, NEC (without/in remission) | C946 (0/1) | - |
| **Other** | Other mature T/NK-cell lymphomas | C845 | - |
|  | Cutaneous T-cell lymphoma, unspecified | C848 | - |
|  | Mature T/NK-cell lymphoma, unspecified | C849 | - |
|  | Extranodal NK/T-cell lymphoma, nasal type | C860 | - |
|  | Hepatosplenic T-cell lymphoma | C861 | - |
|  | Enteropathy-type (intestinal) T-cell lymphoma | C862 | - |
|  | Subcutaneous panniculitis-like T-cell lymphoma | C863 | - |
|  | Blastic NK-cell lymphoma | C864 | - |
|  | Angioimmunoblastic T-cell lymphoma | C865 | - |
|  | Primary cutaneous CD30-positive T-cell proliferations | C866 | - |
|  | Other heavy chain disease (without/in remission) | C882 (0/1) | - |
|  | Immunoproliferative small intestinal disease (without/in remission) | C883 (0/1) | - |
|  | Other malignant immunoproliferative diseases (without/in remission) | C887 (0/1) | - |
|  | Malignant immunoproliferative disease, unspecified (without/in remission) | C889 (0/1) | - |
|  | Plasma cell leukaemia (without/in remission) | C901 (0/1) | - |
|  | Extramedullary plasmacytoma (without/in remission) | C902 (0/1) | - |
|  | Solitary plasmacytoma (without/in remission) | C903 (0/1) | - |
|  | Prolymphocytic leukaemia of B-cell type (without/in remission) | C913 (0/1) | - |
|  | Hairy cell leukaemia (without/in remission) | C914 (0/1) | - |
|  | Adult T-cell leukaemia/lymphoma [HTLV-1-associated] (without/in remission) | C915 (0/1) | - |
|  | Prolymphocytic leukaemia of T-cell type (without/in remission) | C916 (0/1) | - |
|  | Other lymphoid leukaemia (without/in remission) | C917 (0/1) | - |
|  | Mature B-cell leukaemia Burkitt-type (without/in remission) | C918 (0/1) | - |
|  | Lymphoid leukaemia, unspecified (without/in remission) | C919 (0/1) | - |
|  | Atypical chronic myeloid leukaemia, BCR/ABL – negative (without/in remission) | C922 (0/1) | - |
|  | Myeloid sarcoma (without/in remission) | C923 (0/1) | - |
|  | Acute promyelocytic leukaemia (without/in remission) | C925 (0/1) | - |
|  | Other myeloid leukaemia (without/in remission) | C927 (0/1) | - |
|  | Myeloid leukaemia, unspecified (without/in remission) | C929 (0/1) | - |
|  | Acute monoblastic/monocytic leukaemia (without/in remission) | C930 (0/1) | - |
|  | Chronic myelomonocytic leukaemia (without/in remission) | C931 (0/1) | - |
|  | Juvenile myelomonocytic leukaemia (without/in remission) | C933 (0/1) | - |
|  | Other monocytic leukaemia (without/in remission) | C937 (0/1) | - |
|  | Monocytic leukaemia, unspecified (without/in remission) | C939 (0/1) | - |
|  | Acute erythroid leukaemia (without/in remission) | C940 (0/1) | - |
|  | Acute megakaryoblastic leukaemia (without/in remission) | C942 (0/1) | - |
|  | Mast cell leukaemia (without/in remission) | C943 (0/1) | - |
|  | Acute panmyelosis with myelofibrosis (without/in remission) | C944 (0/1) | - |
|  | Other specified leukaemias (without/in remission) | C947 (0/1) | - |
|  | Acute leukaemia of unspecified cell type (without/in remission) | C950 (0/1) | - |
|  | Chronic leukaemia of unspecified cell type (without/in remission) | C951 (0/1) | - |
|  | Other leukaemia of unspecified cell type (without/in remission) | C957 (0/1) | - |
|  | Leukaemia, unspecified (without/in remission) | C959 (0/1) | - |
|  | Multifocal and multisystemic (disseminated) Langerhans-cell histiocytosis [Letterer-Siwe disease] | C960 | - |
|  | Malignant mast cell tumour | C962 | - |
|  | Sarcoma of dendritic cells (accessory cells) | C964 | - |
|  | Multifocal and unisystemic Langerhans-cell histiocytosis | C965 | - |
|  | Unifocal Langerhans-cell histiocytosis | C966 | - |
|  | Other specified malignant neoplasms of lymphoid, haematopoietic and related tissue | C967 | - |
|  | Histiocytic sarcoma | C968 | - |
|  | Malignant neoplasm of lymphoid, haematopoietic and related tissue, unspecified | C969 | - |
|  | Chronic erythraemia (without/in remission) | C941 (0/1) | - |

Abbreviations: ALK, anaplastic large cell kinase; BCR/ABL, breakpoint cluster region protein/Abelson murine leukaemia viral oncogene homolog; CD30, cluster of differentiation 30; HTLV-1, human T-cell leukaemia/lymphoma virus type 1; ICD-10-AM; International Statistics Classification of Diseases and Related Health Problems, Tenth Revision, Australian Modification; MALT, mucosa-associated lymphoid tissue; NEC, not elsewhere classified; NK, natural killer;

**Table 7.** **Invasive Fungal Disease Category Definitions and its Corresponding ICD-10-AM Diagnostic Code, Eighth Edition.**

| **Invasive fungal disease** | **Definition(s)** | **ICD-10-AM diagnostic code** | **Modifications to ICD-10-AM diagnostic codes from fourth to seventh editions** |
| --- | --- | --- | --- |
| **Candidal endocarditis** | Candidal endocarditis | B376 | - |
| **Candidal meningitis** | Candidal meningitis | B375 | - |
| **Candidal sepsis** | Candidal sepsis | B377 | - |
| **Candidiasis of other sites** | Candidiasis of other sites | B3788 | - |
| **Chromomycosis and phaeomycotic abscess** | Chromomycosis, unspecified | B439 | - |
|  | Cutaneous chromomycosis | B430 | - |
|  | Other forms of chromomycosis | B438 | - |
|  | Phaeomycotic brain abscess | B431 | - |
|  | Subcutaneous phaeomycotic abscess and cyst | B432 | - |
| **Cryptococcosis** | Cerebral cryptococcosis | B451 | - |
|  | Cryptococcosis, unspecified | B459 | - |
|  | Cutaneous cryptococcosis | B452 | - |
|  | Disseminated cryptococcosis | B457 | - |
|  | Osseous cryptococcosis | B453 | - |
|  | Other forms of cryptococcosis | B458 | - |
|  | Pulmonary cryptococcosis | B450 | - |
| **Invasive aspergillosis** | Aspergillosis, unspecified | B449 | - |
|  | Invasive pulmonary aspergillosis | B440 | - |
|  | Disseminated aspergillosis | B447 | - |
|  | Other forms of aspergillosis | B448 | - |
|  | Other pulmonary aspergillosis | B441 | - |
|  | Tonsillar aspergillosis | B442 | - |
| **Mucormycosis** | Cutaneous mucormycosis | B463 | - |
|  | Disseminated mucormycosis | B464 | - |
|  | Gastrointestinal mucormycosis | B462 | - |
|  | Mucormycosis, unspecified | B465 | - |
|  | Other zygomycoses | B465 | - |
|  | Pulmonary mucormycosis | B460 | - |
|  | Rhinocerebral mucormycosis | B461 | - |
|  | Zygomycosis, unspecified | B469 | - |
| **Mycetoma** | Actinomycetoma | B471 | - |
|  | Eumycetoma | B470 | - |
|  | Mycetoma, unspecified | B479 | - |
| **Pneumocystosis** | Pneumocystosis | B50 | - |
| **Sporotrichosis** | Disseminated sporotrichosis | B427 | - |
|  | Lymphocutaneous sporotrichosis | B421 | - |
|  | Other forms of sporotrichosis | B428 | - |
|  | Pulmonary sporotrichosis | B420 | - |
|  | Sporotrichosis, unspecified | B429 | - |
| **Other IFD, NOS** | Allescheriasis | B482 | - |
|  | Geotrichosis | B483 | - |
|  | Lobomycosis | B480 | - |
|  | Opportunistic mycoses | B487 | - |
|  | Other specified mycoses | B488 | - |
|  | Penicillosis | B484 | - |
|  | Rhinosporidiosis | B481 | - |
| **Unspecified IFD** | Unspecified mycosis (including fungaemia) | B49 | - |

Abbreviations: ICD-10-AM; International Statistics Classification of Diseases and Related Health Problems, Tenth Revision, Australian Modification; IFD, invasive fungal disease; NOS, not otherwise specified

**Table 8. Procedure Category Definitions and its Corresponding ICD-10-AM Procedural Code, Eighth Edition.**

| **Procedure** | **Definition(s)** | **ICD-10-AM procedural code** | **Modifications to ICD-10-AM procedural codes from fourth to seventh editions** |
| --- | --- | --- | --- |
| **Allogeneic HSCT, matched related donor** | Allogeneic bone marrow or stem cell transplantation, matched related donor, with *in vitro* processing | 13706-06 [802] | - |
|  | Allogeneic bone marrow or stem cell transplantation, matched related donor, without *in vitro* processing | 13706-00 [802] | - |
| **Allogeneic HSCT, other donor** | Allogeneic bone marrow or stem cell transplantation, other donor, with *in vitro* processing | 13706-09 [802] | - |
|  | Allogeneic bone marrow or stem cell transplantation, other donor, without *in vitro* processing | 13706-10 [802] | - |
| **Autologous HSCT** | Autologous bone marrow or stem cell transplantation, with *in vitro* processing | 13706-07 [802] | - |
|  | Autologous bone marrow or stem cell transplantation, without *in vitro* processing | 13706-08 [802] | - |
| **Biopsy of lung** | Biopsy of lung | 38418-02 [550] | - |
|  | Biopsy of pleura | 38418-01 [550] | - |
|  | Percutaneous needle biopsy of lung | 38812-00 [550] | - |
|  | Percutaneous needle biopsy of pleura | 30090-00 [550] | - |
| **Bronchoscopy** | Biopsy of pleura | 38418-01 [550] | - |
|  | Percutaneous needle biopsy of lung | 38812-00 [550] | - |
|  | Percutaneous needle biopsy of pleura | 30090-00 [550] | - |
|  | Excision of bronchogenic cyst via thoracotomy | 43912-00 [545] | - |
|  | Fibreoptic bronchoscopy | 41898-00 [543] | - |
|  | Fibreoptic bronchoscopy with biopsy | 41898-01 [544] | - |
|  | Fibreoptic bronchoscopy with broncho-alveolar lavage [BAL] | 41898-02 [544] | - |
|  | Fibreoptic bronchoscopy with removal of foreign body | 41898-03 [544] | - |
|  | Other endoscopic excision of bronchus | 90163-00 [545] | - |
|  | Rigid bronchoscopy | 41889-00 [543] | - |
|  | Rigid bronchoscopy with biopsy | 41892-00 [544] | - |
|  | Rigid bronchoscopy with removal of foreign body | 41895-00 [544] | - |
| **Chemotherapy** | Administration of pharmacological agent via external vascular catheter – antineoplastic agent | 96204-00 [1920] | - |
|  | Intra-arterial administration of pharmacological agent – antineoplastic therapy | 96196-00 [1920] | - |
|  | Intrathecal administration of pharmacological agent – antineoplastic therapy | 96198-00 [1920] | - |
|  | Intravenous administration of pharmacological agent – antineoplastic therapy | 96199-00 [1920] | - |
|  | Loading of ambulatory drug delivery device – antineoplastic therapy | 96208-00 [1920] | - |
|  | Loading of implantable infusion device or pump – antineoplastic therapy | 96208-00 [1920] | - |
|  | Subcutaneous administration of pharmacological agent – antineoplastic therapy | 96200-00 [1920] | - |
|  | Unspecified administration of pharmacological agent – antineoplastic therapy | 96206-00 [1920] | - |
| **Haemodialysis** | Continuous haemodiafiltration | 13100-04 [1060] | - |
|  | Continuous haemofiltration | 13100-02 [1060] | - |
|  | Haemodialysis | 13100-00 [1060] | - |
|  | Haemoperfusion | 13100-05 [1060] | - |
|  | Intermittent haemodiafiltration | 13100-03 [1060] | - |
|  | Intermittent haemofiltration | 13100-01 [1060] | - |

Abbreviations: BAL, broncho-alveolar lavage; HSCT, haematopoietic stem cell transplantation; ICD-10-AM; International Statistics Classification of Diseases and Related Health Problems, Tenth Revision, Australian Modification

**Table 9.** **Charlson Comorbidity Index Category Definitions and its Corresponding ICD-10-AM Diagnostic Code, Eighth Edition.**

| **Charlson Comorbidity Index condition** | **Definition(s)** | **ICD-10-AM diagnostic code** | **Modifications to ICD-10-AM diagnostic codes from fourth to seventh editions** |
| --- | --- | --- | --- |
| **Acquired immune deficiency syndrome (AIDS)** | Unspecified human immunodeficiency virus [HIV] disease (including AIDS) | B24 | - |
| **Cerebrovascular disease** | Other cerebrovascular diseases – transient cerebral ischaemic attack, unspecified | I67-G459 | - |
| **Chronic pulmonary disease** | Chronic obstructive pulmonary disease with acute exacerbation, unspecified | J441 | - |
|  | Chronic obstructive pulmonary disease with acute lower respiratory infection | J440 | - |
|  | Chronic obstructive pulmonary disease, unspecified | J449 | - |
|  | Other specified chronic obstructive pulmonary disease | J448 | - |
| **Congestive heart failure** | Congestive heart failure | I500 | - |
| **Connective tissue disease** | Behçet’s disease | M352 | - |
|  | Diffuse (eosinophilic) fasciitis | M354 | - |
|  | Hypermobility syndrome | M357 | - |
|  | Multifocal fibrosclerosis | M355 | - |
|  | Other overlap syndromes | M351 | - |
|  | Other specified systemic involvement of connective tissue | M358 | - |
|  | Polymyalgia rheumatica | M353 | - |
|  | Relapsing panniculitis [Weber-Christian] | M356 | - |
|  | Sicca syndrome [Sjögren] | M350 | - |
|  | Systemic involvement of connective tissue, unspecified | M359 | - |
| **Dementia** | Dementia in Alzheimer’s disease | F00 | - |
|  | Dementia in other diseases classified elsewhere | F02 | - |
|  | Unspecified dementia | F03 | - |
|  | Vascular dementia | F01 | - |
| **Diabetes with end-organ damage** | Type 1 diabetes mellitus with acidosis | E101 | - |
|  | Type 1 diabetes mellitus with circulatory complication | E105 | - |
|  | Type 1 diabetes mellitus with hyperosmolarity | E100 | - |
|  | Type 1 diabetes mellitus with kidney complication | E102 | - |
|  | Type 1 diabetes mellitus with multiple complications | E107 | - |
|  | Type 1 diabetes mellitus with neurological complication | E104 | - |
|  | Type 1 diabetes mellitus with other specified complication | E106 | - |
|  | Type 1 diabetes mellitus with other specified kidney complication | E103 | - |
| **Hemiplegia** | Flaccid hemiplegia | G810 | - |
|  | Hemiplegia, unspecified | G819 | - |
|  | Spastic hemiplegia | G811 | - |
| **Leukaemia (acute or chronic) *** | Acute erythroid leukaemia | C940 | - |
|  | Acute leukaemia of unspecified cell type | C950 | - |
|  | Acute lymphoblastic leukaemia [ALL] | C910 | - |
|  | Acute megakaryoblastic leukaemia | C942 | - |
|  | Acute monoblastic/monocytic leukaemia | C930 | - |
|  | Acute myeloblastic leukaemia [AML] | C920 | - |
|  | Acute myelomonocytic leukaemia | C925 | - |
|  | Acute panmyelosis with myelofibrosis | C944 | - |
|  | Acute promyelocytic leukaemia [PML] | C924 | - |
|  | Atypical chronic myeloid leukaemia, BCR/ABL-negative | C922 | - |
|  | Chronic lymphocytic leukaemia of B-cell type | C911 | C912 |
|  | Chronic myeloid leukaemia [CML], BCR/ABL-positive | C921 | - |
|  | Chronic myelomonocytic leukaemia | C931 | - |
|  | Myelodysplastic and myeloproliferative disease, not elsewhere classified | C946 | - |
|  | Other specified leukaemias | C947 | - |
| **Liver disease** | Alcoholic liver disease | K70 | - |
|  | Chronic hepatitis, not elsewhere classified | K73 | - |
|  | Fibrosis and cirrhosis of liver | K74 | - |
|  | Hepatic failure, not elsewhere classified | K72 | - |
|  | Liver disorders in diseases classified elsewhere | K77 | - |
|  | Other diseases of liver | K76 | - |
|  | Other inflammatory liver diseases | K75 | - |
|  | Toxic liver disease | K71 | - |
| **Lymphoma** | Follicular lymphoma | C82 | - |
|  | Hodgkin lymphoma | C81 | - |
|  | Malignant immunoproliferative diseases | C88 | - |
|  | Mature T/NK-cell lymphomas | C84 | - |
|  | Non-follicular lymphoma | C83 | - |
|  | Other and unspecified types of non-Hodgkin lymphoma | C85 | - |
|  | Other specified types of T/NK-cell lymphoma | C86 | - |
| **Metastatic solid tumour** | Malignant neoplasm of other and ill-defined sites | C76 | - |
|  | Malignant neoplasms, stated or presumed to be primary, of specified sites, except of lymphoid haematopoietic and related tissue | C00 – C75 | - |
|  | Malignant neoplasm without specification of site | C80 | - |
|  | Secondary malignant neoplasm of other and unspecified sites | C79 | - |
|  | Secondary malignant neoplasm of respiratory and digestive organs | C78 | - |
| **Moderate or severe liver disease** | Alcoholic liver disease | K70 | - |
|  | Chronic hepatitis, not elsewhere classified | K73 | - |
|  | Fibrosis and cirrhosis of liver | K74 | - |
|  | Hepatic failure, not elsewhere classified | K72 | - |
|  | Liver disorders in diseases classified elsewhere | K77 | - |
|  | Other diseases of liver | K76 | - |
|  | Other inflammatory liver diseases | K75 | - |
|  | Toxic liver disease | K71 | - |
| **Moderate or severe renal disease** | Acute kidney failure | N17 | - |
|  | Chronic kidney failure | N18 | - |
|  | Unspecified kidney failure | N19 | - |
| **Myocardial infarction** | Acute myocardial infarction, unspecified | I219 | - |
|  | Acute transmural myocardial infarction of anterior wall | I210 | - |
|  | Acute transmural myocardial infarction of inferior wall | I211 | - |
|  | Acute transmural myocardial infarction of other sites | I212 | - |
|  | Acute transmural myocardial infarction of unspecified site | I213 | - |
|  | Acute subendocardial myocardial infarction | I214 | - |
| **Peptic ulcer disease** | Peptic ulcer, site unspecified | K27 | - |
| **Peripheral vascular disease** | Aortic aneurysm and dissection | I71 | - |
|  | Atherosclerosis of arteries of extremities | I702 | - |
|  | Other aneurysm and dissection | I72 | - |
|  | Other peripheral vascular diseases | I73 | - |
| **Tumour without metastases** | Benign lipomatous neoplasm | D17 | - |
|  | Benign neoplasm of bone and articular cartilage | D16 | - |
|  | Benign neoplasm of brain and other parts of central nervous system | D33 | - |
|  | Benign neoplasm of breast | D24 | - |
|  | Benign neoplasm of colon, rectum, anus and anal canal | D12 | - |
|  | Benign neoplasm of eye and adnexa | D31 | - |
|  | Benign neoplasm of major salivary glands | D11 | - |
|  | Benign neoplasm of male genital organs | D29 | - |
|  | Benign neoplasm of meninges | D32 | - |
|  | Benign neoplasm of mesothelial tissue | D19 | - |
|  | Benign neoplasm of middle ear and respiratory system | D14 | - |
|  | Benign neoplasm of mouth and pharynx | D10 | - |
|  | Benign neoplasm of other and unspecified female genital organs | D28 | - |
|  | Benign neoplasm of other and ill-defined parts of digestive system | D13 | - |
|  | Benign neoplasm of other and unspecified endocrine glands | D35 | - |
|  | Benign neoplasm of other and unspecified intrathoracic organs | D15 | - |
|  | Benign neoplasm of other and unspecified sites | D36 | - |
|  | Benign neoplasm of ovary | D27 | - |
|  | Benign neoplasm of soft tissue of retroperitoneum and peritoneum | D20 | - |
|  | Benign neoplasm of urinary organs | D30 | - |
|  | Benign neoplasm of thyroid gland | D34 | - |
|  | Haemangioma and lymphangioma | D18 | - |
|  | Leiomyoma of uterus | D25 | - |
|  | Melanocytic naevi | D22 | - |
|  | Other benign neoplasms of connective and other soft tissue | D21 | - |
|  | Other benign neoplasm of skin | D23 | - |
|  | Other benign neoplasms of uterus | D26 | - |

Abbreviations: AIDS, acquired immune deficiency syndrome; ALL, acute lymphoblastic leukaemia; AML, acute myeloblastic leukaemia; BCR/ABL, breakpoint cluster region protein/Abelson murine leukaemia viral oncogene homolog; CML, chronic myeloid leukaemia; ICD-10-AM; International Statistics Classification of Diseases and Related Health Problems, Tenth Revision, Australian Modification; HIV, human immunodeficiency virus; NK, natural killer; PML, promyelocytic leukaemia
