# Supplementary material for: A population-based analysis of invasive fungal disease in haematology-oncology patients using data linkage of state-wide registries and administrative databases: 2005 - 2016
Source: BMC Infect Dis. 2019 Mar 21;19:274. doi: 10.1186/s12879-019-3901-y (PMC6429824; doi:10.1186/s12879-019-3901-y)
Supplement: Supplementary file 3 — : Exclusion ICD-10-AM Codes. Administrative coding data used to identify superficial fungal infections that were excluded from this study. (DOCX 14 kb) [file 12879_2019_3901_MOESM3_ESM.docx]

**Additional File 3**

Exclusion codes

Fungal diseases omitted from the study:

Fungal infections endemic to parts of North America as follows:

- Coccidioidomycoses (ICD-10-AM diagnostic code: B38)
- Paracoccidioidomycoses (ICD-10-AM diagnostic code: B41)
- Histoplasmosis (ICD-10-AM diagnostic code: B39)
- Blastomycoses (ICD-10-AM diagnostic code: B40)

and superficial fungal infections as follows:

- Candidal stomatitis (ICD-10-AM diagnostic code: B370)
- Candidiasis of the skin and nail (ICD-10-AM diagnostic code: B372)
- Candidiasis of the vulva and vagina (ICD-10-AM diagnostic code: B373)
- Candidiasis of other urogenital sites (ICD-10-AM diagnostic code: B374)
- Candidiasis, unspecified (ICD-10-AM diagnostic code: B379)
- Dermatophytosis (ICD-10-AM diagnostic code: B35)

The before-mentioned fungal infections are not invasive and were thus omitted from the study.
