# Supplementary material for: A population-based analysis of invasive fungal disease in haematology-oncology patients using data linkage of state-wide registries and administrative databases: 2005 - 2016
Source: BMC Infect Dis. 2019 Mar 21;19:274. doi: 10.1186/s12879-019-3901-y (PMC6429824; doi:10.1186/s12879-019-3901-y)

**Additional File 4**

Receiver operating curve (ROC) and its corresponding C-statistic for the evaluation of the multivariable logistic regression model


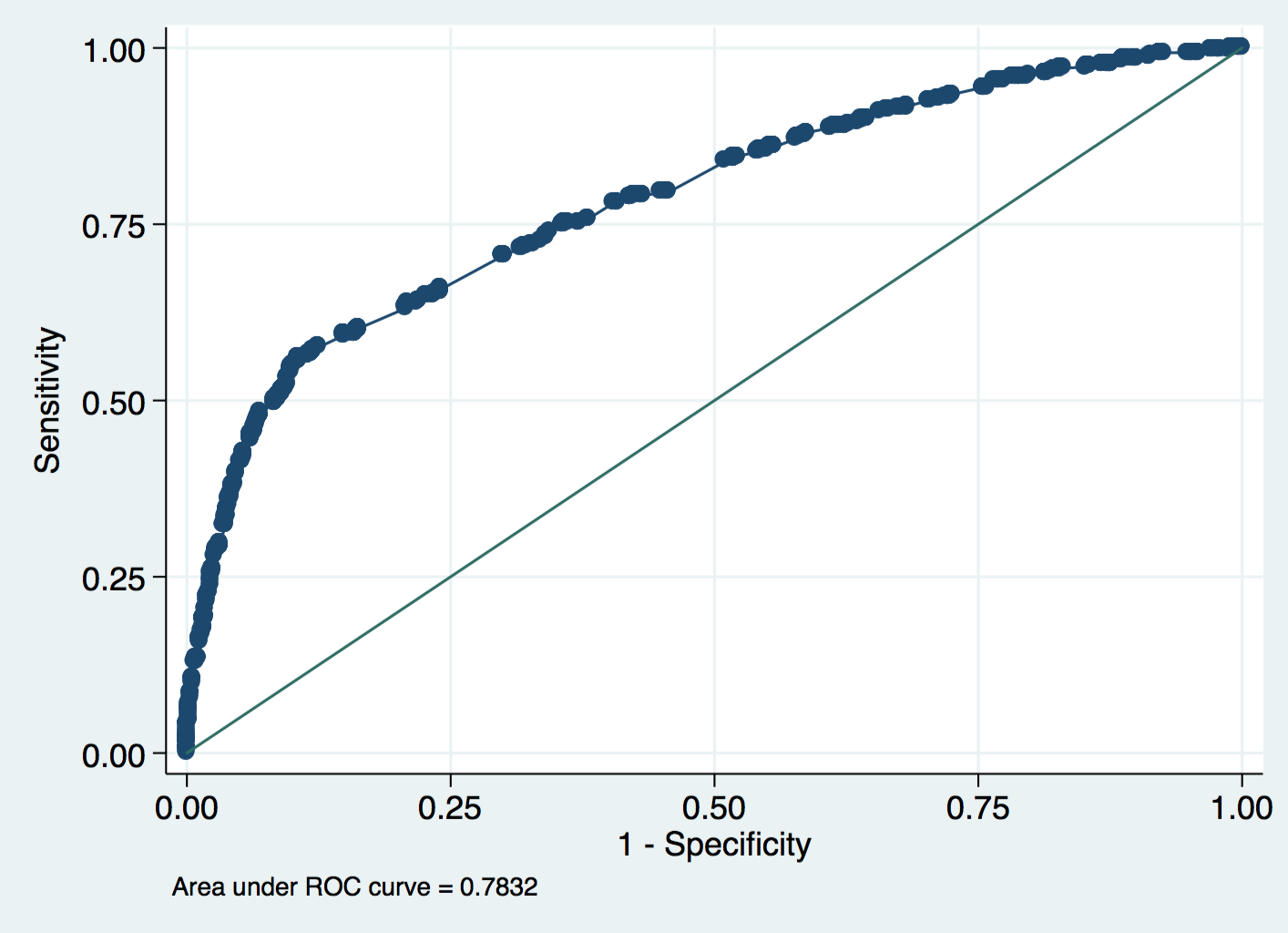

Supplement: Supplementary file 4 — Receiver Operating Curve and C-Statistic. Receiver operating curve and its corresponding C-statistic for the evaluation of the multivariable logistic regression model. (DOCX 162 kb) [file 12879_2019_3901_MOESM4_ESM.docx]
