# Supplementary material for: A population-based analysis of invasive fungal disease in haematology-oncology patients using data linkage of state-wide registries and administrative databases: 2005 - 2016
Source: BMC Infect Dis. 2019 Mar 21;19:274. doi: 10.1186/s12879-019-3901-y (PMC6429824; doi:10.1186/s12879-019-3901-y)
Supplement: Supplementary file 5 — Distribution of time (months) to invasive fungal disease among allogeneic haematopoietic stem cell transplantation (HSCT) recipients stratified by graft-versus-host disease (GVHD) status post-transplantation (GVHD negative, N=13; GVHD positive, N=28). Box-plot stratified by allogeneic-HSCT recipients with and without GVHD detailing the median time (in months) to invasive fungal disease onset. (DOCX 184 kb) [file 12879_2019_3901_MOESM5_ESM.docx]

**(*Additional File 5)*.** Distribution of time (months) to invasive fungal disease among allogeneic haematopoietic stem cell transplantation (HSCT) recipients stratified by graft-versus-host disease (GVHD) status post-transplantation (GVHD negative, N=13; GVHD positive, N=28).

**
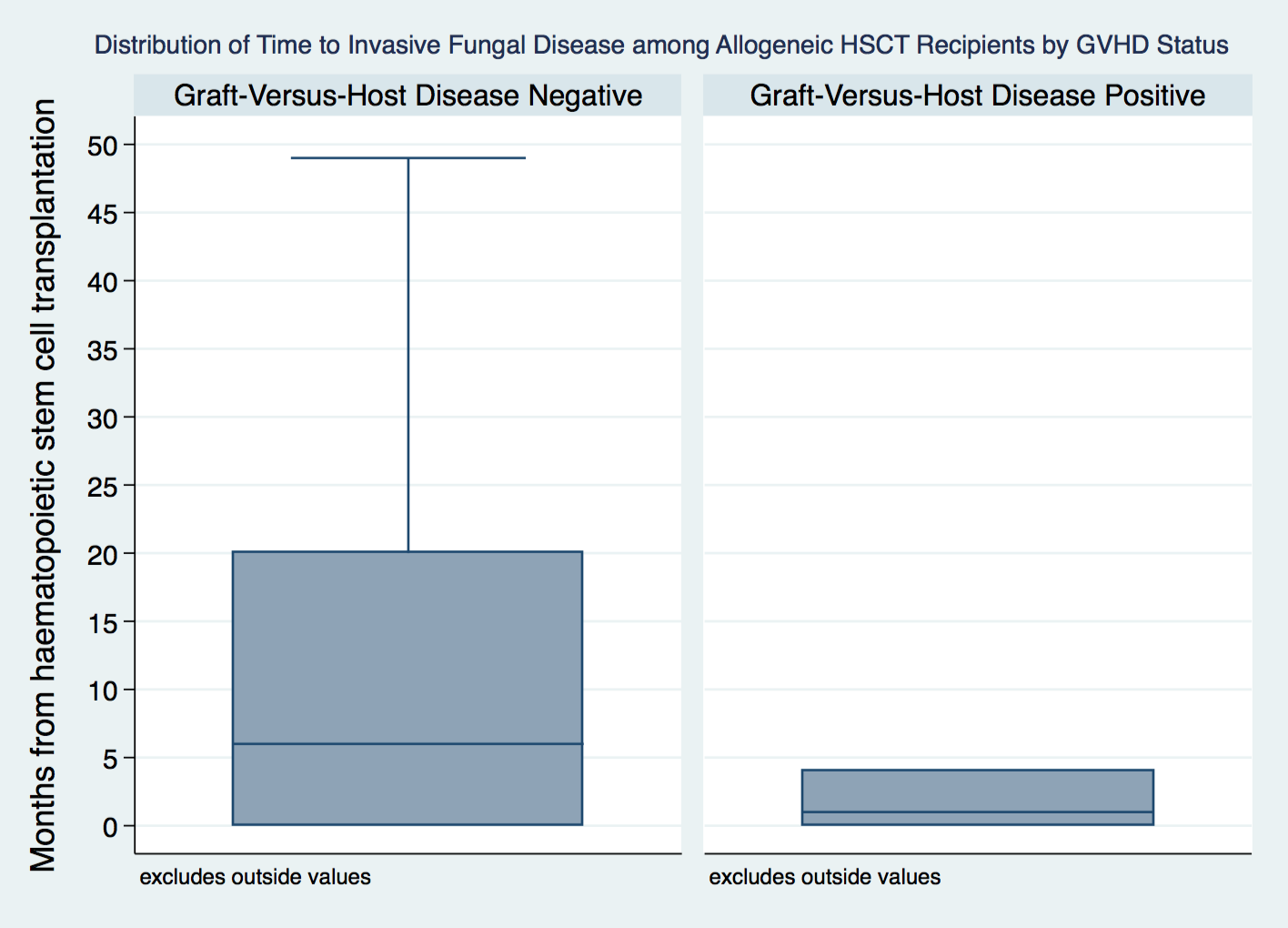
**
